# Supplementary material for: Probing PAC1 receptor activation across species with an engineered sensor
Source: eLife. 2024 Aug 15;13:RP96496. doi: 10.7554/eLife.96496 (PMC11326774; doi:10.7554/eLife.96496)
Supplement: Supplementary file 1. [file elife-96496-supp1.docx]

**PACLight1_P78A_ DNA sequence:**

ATGAAGACGATCATCGCCCTGAGCTACATCTTCTGCCTGGTGTTCGCCATGGCGGGCGTAGTGCACGTTTCCCTTGCTGCACTGCTCCTCTTGCCAATGGCGCCAGCCATGCACAGTGACTGCATCTTTAAGAAGGAACAAGCTATGTGTCTCGAAAAAATCCAAAGAGCTAATGAGCTTATGGGCTTTAATGATTCTTCACCAGGTTGCCCGGGTATGTGGGATAACATTACATGTTGGAAGCCCGCGCACGTAGGTGAAATGGTATTGGTTTCTTGCGCTGAACTGTTCCGAATTTTTAACCCCGATCAAGTATGGGAAACGGAAACGATTGGTGAAAGTGACTTCGGAGACTCAAATAGTCTGGATCTCTCAGATATGGGTGTGGTCTCTCGGAATTGTACCGAGGATGGCTGGTCCGAGCCGTTTCCGCATTATTTTGACGCATGTGGTTTCGACGAATACGAATCTGAGACCGGAGATCAAGATTACTACTACCTTAGTGTAAAGGCACTGTATACAGTTGGTTACAGTACAAGTCTGGTTACACTCACTACAGCTATGGTAATCTTGTGCAGATTCAGGAAGCTCCATTGTACGCGAAATTTCATTCACATGAATTTGTTCGTGTCTTTTATGCTCCGGGCTATTTCCGTTTTCATTAAAGACTGGATTCTGTACGCAGAGCAGGATTCAAACCATTGTTTCATCAGTACAGTAGAATGTAAGGCAGTAATGGTTTTTTTTCATTACTGCGTGGTTTCCAACTACTTTTGGCTGTTTATAGAAGGTTTGTATCTGTTCACACTTCTGGTTGAAACTAAGAAGAAGGAGCGACGGTACTTCTACTGGTACACAATTATAGGATGGGGCACTCCGACCGTCTGTGTGACAGTATGGGCGACACTCCGGCTTTACTTCGACGACACAGGTTGTTGGGACATGAACGATTCCACGGCTCTCTGGTGGGTAATAAAGGGGCCAGTCGTGGGGAGCATTATGGTTAATTTTGTCCTCTTTATCGGCATTATAGTAATCCTTGTCCAGAAGCTGCAGCTGAGCTCACTCATTAACGTCTATATCAAGGCCGACAAGCAGAAGAACGGCATCAAGGCGAACTTCAAGATCCGCCACAACATCGAGGACGGCGGCGTGCAGCTCGCCTACCACTACCAGCAGAACACCCCCATCGGCGACGGCCCCGTGCTGCTGCCCGACAACCACTACCTGAGCGTGCAGTCCAAACTTTCGAAAGACCCCAACGAGAAGCGCGATCACATGGTCCTGCTGGAGTTCGTGACCGCCGCCGGGATCACTCTCGGCATGGACGAGCTGTACAAGGGCGGTACCGGAGGGAGCATGGTGAGCAAGGGCGAGGAGCTGTTCACCGGGGTGGTGCCCATCCTGGTCGAGCTGGACGGCGACGTAAACGGCCACAAGTTCAGCGTGTCCGGCGAGGGTGAGGGCGATGCCACCTACGGCAAGCTGACCCTGAAGTTCATCTGCACCACCGGCAAGCTGCCCGTGCCCTGGCCCACCCTCGTGACCACCCTGACCTACGGCGTGCAGTGCTTCAGCCGCTACCCCGACCACATGAAGCAGCACGACTTCTTCAAGTCCGCCATGCCCGAAGGCTACATCCAGGAGCGCACCATCTTCTTCAAGGACGACGGCAACTACAAGACCCGCGCCGAGGTGAAGTTCGAGGGCGACACCCTGGTGAACCGCATCGAGCTGAAGGGCATCGACTTCAAGGAGGACGGCAACATCCTGGGGCACAAGCTGGAGTACAACAATCATGACCAACTGAATGAGAGTTCTATATACTTGCGCCTCGCGCGAAGTACACTCCTGCTGATCCCTCTTTTTGGTATACATTACACAGTCTTCGCGTTTAGTCCAGAGAACGTCAGTAAACGCGAAAGACTCGTCTTCGAACTGGGGTTGGGTTCATTTCAGGGGTTCGTCGTTGCAGTACTTTATTGCTTTCTGAATGGCGAAGTCCAAGCAGAAATTAAGCGGAAGTGGCGCTCTTGGAAGGTCAATCGCTATTTCGCCGTGGACTTCAAACATCGCCATCCCAGCTTGGCTTCCTCAGGCGTGAATGGGGGCACGCAGTTGAGTATTCTGTCAAAGAGTTCATCCCAGATTAGAATGTCAGGTCTCCCGGCCGACAACTTGGCCGCATGA

**PACLight1_P78A_ protein sequence:**

MKTIIALSYIFCLVFAMAGVVHVSLAALLLLPMAPAMHSDCIFKKEQAMCLEKIQRANELMGFNDSSPGCPGMWDNITCWKPAHVGEMVLVSCAELFRIFNPDQVWETETIGESDFGDSNSLDLSDMGVVSRNCTEDGWSEPFPHYFDACGFDEYESETGDQDYYYLSVKALYTVGYSTSLVTLTTAMVILCRFRKLHCTRNFIHMNLFVSFMLRAISVFIKDWILYAEQDSNHCFISTVECKAVMVFFHYCVVSNYFWLFIEGLYLFTLLVETKKKERRYFYWYTIIGWGTPTVCVTVWATLRLYFDDTGCWDMNDSTALWWVIKGPVVGSIMVNFVLFIGIIVILVQKLQLSSLINVYIKADKQKNGIKANFKIRHNIEDGGVQLAYHYQQNTPIGDGPVLLPDNHYLSVQSKLSKDPNEKRDHMVLLEFVTAAGITLGMDELYKGGTGGSMVSKGEELFTGVVPILVELDGDVNGHKFSVSGEGEGDATYGKLTLKFICTTGKLPVPWPTLVTTLTYGVQCFSRYPDHMKQHDFFKSAMPEGYIQERTIFFKDDGNYKTRAEVKFEGDTLVNRIELKGIDFKEDGNILGHKLEYNNHDQLNESSIYLRLARSTLLLIPLFGIHYTVFAFSPENVSKRERLVFELGLGSFQGFVVAVLYCFLNGEVQAEIKRKWRSWKVNRYFAVDFKHRHPSLASSGVNGGTQLSILSKSSSQIRMSGLPADNLAA*

Color legend:

HA secretory sequence

hmPAC1R secretory sequence

Mutations added during optimization

cpGFP
